# CSgator: an integrated web platform for compound set analysis

**DOI:** 10.1186/s13321-019-0339-6

**Published:** 2019-03-04

**Authors:** Sera Park, Yeajee Kwon, Hyesoo Jung, Sukyung Jang, Haeseung Lee, Wankyu Kim

**Affiliations:** 1 Ewha Research Center for Systems Biology, Department of Life Science, Division of Molecular and Life Sciences, Ewha Womans University, Seoul, Korea; 2 KaiPharm, Seoul, Korea

**Keywords:** Compound set analysis, Drug target, Bioactivity profile, Bioassay, Compound network

## Abstract

Drug discovery typically involves investigation of a set of compounds (e.g. drug screening hits) in terms of target, disease, and bioactivity. CSgator is a comprehensive analytic tool for set-wise interpretation of compounds. It has two unique analytic features of *Compound Set Enrichment Analysis* (CSEA) and *Compound Cluster Analysis* (CCA), which allows batch analysis of compound set in terms of (i) target, (ii) bioactivity, (iii) disease, and (iv) structure. CSEA and CCA present enriched profiles of targets and bioactivities in a compound set, which leads to novel insights on underlying drug mode-of-action, and potential targets. Notably, we propose a novel concept of ‘*Hit Enriched Assays*”, i.e. bioassays of which hits are enriched among a given set of compounds. As an example, we show its utility in revealing drug mode-of-action or identifying hidden targets for anti-lymphangiogenesis screening hits. *CSgator* is available at http://csgator.ewha.ac.kr, and most analytic results are downloadable.

## Introduction

During the early phase of drug discovery, it is common to identify multiple hit compounds by high-throughput screening (HTS) [1, 2]. It is critical to survey their known targets, activities, and disease indications to avoid potential toxicity or side-effects, to understand structure-activity relations (SAR), and to direct medicinal chemistry for lead generation. Active exploitation of polypharmacology (e.g. dual inhibitors) or drug combination has also been considered as a viable strategy to overcome drug resistance or tumor heterogeneity in cancer therapy [3]. Although scientists have access to many chemogenomic databases, they are not comprehensive enough individually, nor suitable or convenient for batch analyses of a compound set [4–8]. Recent explosion of bioassay datasets (e.g. PubChem and ChEMBL [9, 10]) made rich information available on diverse aspects of bioactivities, but such data have been used only limitedly in drug discovery. Several integrated compound-target DBs are available, but are limited in analytic functions [11, 12]. There were several works on predictive analyses based on bioactivity profiles or fingerprints, most of which did not fully exploited bioactivity data available [13, 14], or were difficult to use for researchers without programming skills [15].

Here, we developed CSgator (Compound Set navigator), a web platform that provides a comprehensive interpretation of compound set. It is equipped with unique analytic features of *Compound Set Enrichment Analysis* (CSEA) and *Compound Cluster Analysis* (CCA). Particularly, we provide unique analytic functions such as *HEA* analysis (*Hit Enrichment Analysis*) that provide novel insights or clues on drug mode-of-action, or underlying targets of phenotypic screening hits (e.g. lymphangiogenesis) as described in the following sections with an example case.

## Materials and methods

### Standardization of compound IDs and gene names

In order to avoid redundancy, CSgator amassed a consolidated set of compounds from public chemical database such as PubChem, ChEMBL, ChEBI, and DrugBank [5, 7, 10, 16]. We then merged different isotopic, (un)charged, and (de)protonated forms of the same molecule into a single compound ID. For example, lovastatin, a HMG-CoA reductase inhibitor falls into 62 PubChem CIDs, all of which would show essentially the same or highly similar biological activity. All the compounds were mapped to a unified compound ID based on IUPAC InChIKey (IUPAC International Chemical Identifier Key) using Open Babel v.2.3 [17] by converting SMILES or MOL format to InChIKey strings as well as by manual mapping of compound names where necessary. Gene IDs were standardized using the gene names given by UniProtKB and NCBI Gene [18, 19].

### Collection of compound-target interaction data

We collected compound-target interaction data from 15 public databases: CTD, DCDB, DrugBank, MATADOR, TTD, BindingDB, ChEMBL, KiDB, KEGG Drug, PharmGKB, IUPHAR, Binding MOAD, DGIdb, GLASS, STITCH [4, 6–8, 10, 20–29]. After ID standardization of compounds and genes, a total of > 3 mil. compound-target interactions are collected (Table 1).

**Table 1:** Compound-target interaction data from 15 public databases

Source name	# interactions
BindingDB	1,078,520
Binding MOAD	15,320
Comparative Toxicogenomics Database	77,327
ChEMBL v.21	512,341
DCDB	1902
DGIdb	16,852
DrugBank	12,501
IUPHAR	12,429
KEGG Drug	9787
KiDB	20,610
MATADOR	1163
PharmGKB	3606
Therapeutic Targets Database	45,901
GLASS	460,881
STITCH v.5^a^	788,024
Total	3,057,164

^a^STITCH provides scores for protein–chemical interactions, we filtered that interactions on two conditions: experimental score ≥ 700 and database score ≥ 700

### Classification of targets and diseases

Compounds and targets were classified by four different annotations: (I) Protein family classes by ChEMBL version 21, (II) Gene Ontology (GO) terms on Biological Process (BP) [30], (III) Disease Ontology (DO) terms that cross reference with MeSH (Medical Subject Headings), ICD (International Classification of Diseases), NCI’s thesaurus, SNOMED (Systemized Nomenclature of Medicine) and OMIM [31], and (IV) MeSH Disease term provided by NLM (U.S. National Library of Medicine) [32].

### Bioassay data from PubChem Bioassay and ChEMBL

Bioassay data include information for diverse aspects of compound bioactivities. We collected over 1.2 mil. bioassay dataset for > 2 mil. compounds from PubChem Bioassay and ChEMBL [9, 10]. Some of the bioassay dataset were not in a standardized format and required further processing such as ordering compounds by activity, assignment of hit/non-hit compounds, and target ID standardization for targeted bioassays. We assigned compounds as hit by applying one of the three criteria. First, PubChem Bioassay and ChEMBL provide active/inactive information for ~ 22% of the total assays (~ 270,000 bioassays), and accordingly, we took the information to assign hit or non-hit compounds. For the remaining bioassays without active/inactive annotation, the cut-off of Z score ≥ 2 or the top 1% were applied as the second and the third criteria, and took the union of the resulting compound sets as hits. Because only a small fraction of the assays were annotated to a specific target, we also performed a manual curation to assign bioassays to a specific target whenever target information is available in the assay title or description. As a result, ~ 10.3% of the total assays were assigned to a specific target.

### Generation of structural properties

We calculated structural and physicochemical properties of all the compounds, which can be exploited for characterization or filtering of a compound set using Open Babel toolbox [17]. The physicochemical properties were calculated such as molecular weight, FP2 fingerprint, logP (Partition coefficient), topological polar surface area (TPSA), and hydrogen bond donor and acceptor. Additionally, we generate predictors for lead-likeness, i.e. Lipinski’s the rule, and QED (quantitative estimation of drug-likeness) by Gregory Gerebtzoff (Roche, Switerland) implemented in Silico-it package [33].

## Utility and discussion

### System overview

CSgator includes information on ~ 90 million compounds after merging redundant entries, > 6 million compound-target relations from 15 public databases (Table 1), ~ 1.6 million compound-disease associations, and > 230 million bioactivity points collected from > 1.2 million bioassay data set. Whenever available, compounds and targets were annotated by protein family, functional annotation by Gene Ontology [30], and disease categories by Disease Ontology and MeSH [31, 32]. As described in the following sections, these annotations are crucial to interpret the characteristics of input compound set, and provide novel clues on drug mode-of-action, and will be expanded as more information accumulate. Data sources and current statistics are listed in Table 2. These data may be available elsewhere, but CSgator is unique for its comprehensiveness, clean mapping between different resources, and full data accessibility.

**Table 2:** Data sources and statistics collected in CSgator

	Number of entries	Number of compounds	Sources	Number of relations	Standard ID
*Compound database*					
Compound	–	89,602,599	PubChem, ChEMBL, ChEBI, DrugBank	–	InChIKey
*Compound*-*target & disease & bioassay*					
Target	252,498	852,375	15 Public DBs	6,027,120	Entrez Gene ID & UniProtKB
Disease	5680	10,975	CTD	1,575,457	MeSH & OMIM
Bioassay	1,218,658	2,253,835	PubChem, ChEMBL	229,842,265	PubChem AID & ChEMBL
*Classification*					
Protein family	575	833,590	ChEMBL 21	1,691,879	ChEMBL protein class
GO term	19,234	851,359	Gene Ontology	68,331,986	GO term
Disease ontology	1824	5429	Disease Ontology	46,053	DO term
MeSH disease	6351	6909	NIH	143,277	MeSH
Approval status	9	3765	DrugBank ChEMBL NCGC	12,820	InChIKey

**Fig. 1:**
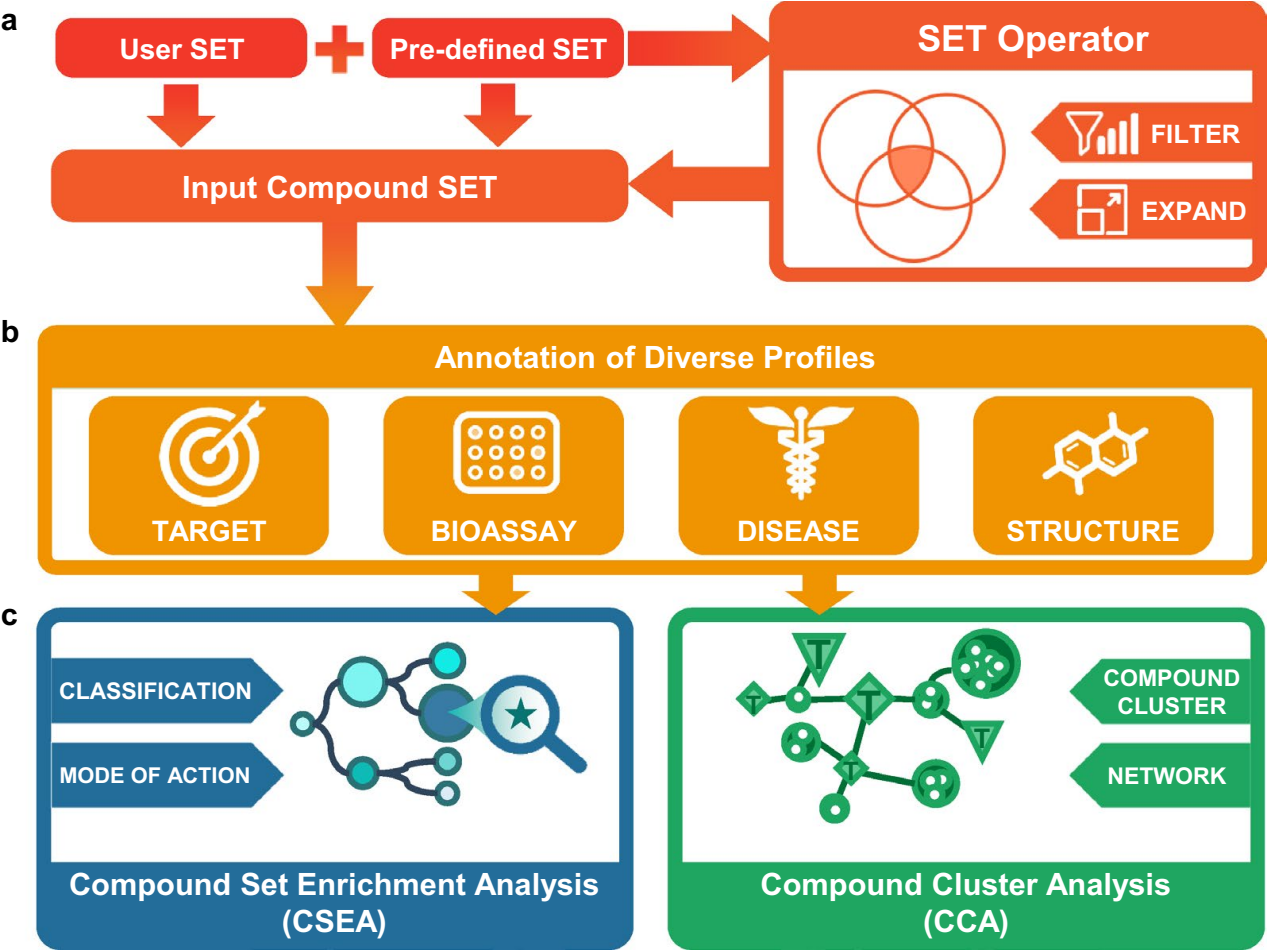
System overview of CSgator web platform. **a** *Input Compound Set* generated by user or selected among the predefined sets. It can be also created by applying various filters, and combining multiple sets using *Set Operator* such as union or intersection. **b** Comprehensive annotations of the input compound set are listed in four categories: *target*, *bioactivity (bioassay)*, *disease*, and *structure*. **c** CSgator provides unique analyses. i.e. Compound Set Enrichment Analysis (CSEA) and Compound Cluster Analysis (CCA), of which details are described in the main text

The analytic workflow of CSgator consists of three steps of (i) generation of input compound set, (ii) tabular listing of annotations for the input compound set, and (iii) compound set analysis step as depicted in Fig. 1. First, input compound set can be generated in three different ways: (a) by *Compound ID Search* using SMILES, InChI, InChIKey, CAS Registry Number, and other IDs including PubChem, ChEMBL, ChEBI, and DrugBank, (b) by *Compound Structure Search* for compounds with specific scaffolds or by structural similarity, and (c) by *Compound Set Selection*, where the precompiled compound set is selected. Precompiled compound sets were built in various ways, e.g. by target or target family, approval status by FDA and other countries, and disease indication. Notably, users can also freely generate a new compound set by applying *Set Operator* to precompiled or input compound sets, and by filtering compounds based on physicochemical properties. Second, CSgator internally gathers all the annotations of input compounds that are grouped into four categories: (i) target, (ii) bioassay, (iii) disease, and (iv) structure. All the annotations are listed and downloadable in a tabular format. Third, user can investigate collective information of a compound set, which is not available in other related databases. The two unique analyses in CSgator are *CSEA (Compound Set Enrichment Analysis)* and *CCA (Compound Cluster Analysis)*, which will be further explained in the following sections.

### Compound Set Enrichment Analysis (CSEA)

Similarly to *Gene Set Enrichment Analysis (GSEA* [34]), *Compound Set Enrichment Analysis (CSEA)* refers to investigating enriched annotations for a compound set. Varin et al. [35] applied CSEA to identify active scaffolds enriched in primary screening data. We extend CSEA even further to annotations on target, disease, and bioassay hits. Particularly, we propose a novel concept of *Hit Enriched Assays (HEAs)* as bioassays of which hits are enriched among the compound set of interest. Since bioassays generally have intended targets and biological processes, HEAs can provide non-obvious links to the underlying targets and drug mode-of-actions enriched in the input compound set such as phenotypic screening hits. Similarly, it also shows enriched targets or diseases in a tree format, i.e. Target Enrichment Tree (TET), and Disease Enrichment Tree (DET). The degree of enrichment, or *Enrichment Score (ES)* is calculated as log likelihood ratio (LLR) for HEAs, and odds ratio for TET and DET.

$$\begin{aligned} & ES_{HEA} = LLR = \log_{2} \left( {\frac{{|Q \cap H|/|Q^{C} \cap H|}}{{|Q|/|Q^{C} |}}} \right) \\ & ES_{TET\,or\,DET} = Odds\,Ratio = \log_{2} \left( {\frac{{|Q \otimes H|/|Q^{C} \otimes H|}}{{|Q \otimes H^{C} |/|Q^{C} \otimes H^{C} |}}} \right) \\ & |Q \otimes H|:The\,number\,of\,interactions\,between\,Q\,(compound\,set)\,and\,H\,(target\,or\,disease), \\ \end{aligned}$$

where Q is the input or query compounds, and Q^C^ is the compounds that do not belong to Q. H is the compounds of interest, e.g. hit compounds for HEA, ligands for a target or target family for TET, and compounds related to a disease for DET analysis. In calculating ES for TET (or DET), we assume compounds not in the query Q^C^ do NOT interact with the target or target family (or disease) although more interactions may exist, but not yet discovered in any test. These missing information may skew the results of TET (or DET), which should be cautiously interpreted. Later, we show an example case of CSEA in interpreting anti-lymphangiogenesis screening hits in the ‘Case Study’ section below.

### Compound Cluster Analysis (CCA)

Structurally similar compounds tend to share the same or structurally similar targets [36]. With the purpose to investigate this aspect, CSgator first generates *Compound Clusters* (CCs) of structurally similar subgroups by k-means clustering. It then shows *Compound Cluster Network* (CC-Network), showing connections among the compound clusters with target family or disease classes. Similarly to CSEA, the degree of enrichment for each CC is also calculated as odd ratio, where R represent the compounds of each cluster (CC), and R^C^ is the all other compounds in the database. Therefore, CC-Network provides information on how a structurally similar cluster of compounds (CC) would be significantly associated to a specific target family or disease class compared to all other compounds as background.

$$\begin{aligned} & {\text{ES}}_{CCA} = {\text{Odd}}\,{\text{Ratio}} = \log_{2} \left( {\frac{{|R \otimes H|/|R^{C} \otimes H|}}{{|R \otimes H^{C} |/|R^{C} \otimes H^{C} |}}} \right) \\ & \left| {R \otimes H} \right|:The\,number\,of\,interactions\,between\,R\,(compound\,cluster)\,and\,H\,(target\,or\,disease) \\ \end{aligned}$$

### A case study on interpreting phenotypic screening hits

Here, we show the utility of the two main analytic functions in CSgator using a case study in interpreting phenotypic screening hits. We took the high-content phenotype-based assay data for screening inhibitors of lymphangiogenesis [37]. Schulz et al. screened FDA approved 1280 drugs (Library of Pharmacologically Active Compounds or LOPAC library from Sigma), resulting in identifying 31 hits (hit rate of 2.4%). The 31 hits were mapped to 40 unique compound IDs in CSgator. But this screening dataset alone does not provide information on the underlying targets or drug mode-of-action. With the 40 compounds as an input set, we performed CSEA and CCA analyses implemented in CSgator as described in the following section.

#### CSEA (Compound Set Enrichment Analysis)

CSEA investigates enriched annotations in terms of target, disease, and bioactivities. In HEA analysis, CSgator listed 146 bioassays, where the 40 input compounds were significantly enriched as hits. We took the list of HEAs that have explicit information on their intended targets with high enrichment score (ES > 5) as listed in Table 3. The targets of the top ranked HEAs include many genes that were known to be involved in lymphangiogenesis. The top ranking HEA (ES = 9.75) screened for RGS4 (Regulator of G-protein signaling 4) inhibitors. Indeed, RGS4 plays a key role in regulating tubulogenesis including lymphangiogenesis by antagonizing MAPK and VEGF signaling [38, 39]. The third and fifth HEA targeted mTOR (ES = 7.12), which generally known to control lymphangiogenesis [40, 41]. Thrombopoietin (TPO) is the regulator of thrombocyte production, and recent studies provide evidence for the critical role of the thrombocytes in lymphangiogenesis in human malignant tumors [42, 43]. A bioassay targeting TPO was ranked at the top 7th with ES = 6.21. Vascular endothelial growth factor D (VEGF-D) has been implicated in the key role of lymphangiogenesis. TNF-α induces AP-1 binding to the VEGF-D promoter, and increase VEGF-D expression through TNF-α/ERK1/2/AP-1 pathway, which promotes lymphangiogenesis and lymphatic metastasis [44, 45]. The 10th HEA (ES = 5.85) targeted AP1 signaling. In summary, five out of the top 10 HEAs provided direct links to the known genes associated to lymphangiogenesis. Accordingly, other targets of high ranking HEAs may be also involved in lymphangiogenesis, such as GMNN, ATAD5, ATXN2, and FEN1 (Table 3).

**Table 3:** HEAs (Hit Enriched Assays) from lymphangiogenesis hits

Rank	Assay title	Target gene	Enrichment score of HEA	Number of hit/assayed compounds	FDR-adjusted *p* value	PubChem AID (year)	Reference
1	Inhibitors of regulator of G protein signaling (RGS) 4	RGS4	9.63	152/390,220	3.40E−16	504,845 (2011)	[38, 39]
2	Validation screen for inhibitors of Lassa infection	–	7.18	54/1279	3.04E−13	463,096 (2010)	
3	High content imaging cell-Based qHTS for inhibitors of the mTORC1 signaling pathway in MEF (Tsc2-/-, p53-/-) cells	MTOR	7.12	23/1280	8.03E−09	2666 (2010)	[40, 41]
4	Validation screen for small molecules that induce DNA re-replication in MCF 10A normal breast cells	GMNN	6.77	71/1280	4.61E−11	463,097 (2010)	
5	High content imaging cell-based qHTS for inhibitors of the mTORC1 signaling pathway in MEF cells	MTOR	6.35	52/1280	1.85E−10	2667 (2010)	[40, 41]
6	Validation screen for small molecules that inhibit ELG1-dependent DNA repair in human embryonic kidney (HEK293T) cells expressing luciferase-tagged ELG1	ATAD5	6.22	79/1280	3.78E−10	493,107 (2011)	
7	qHTS assay for identification of small molecule antagonists for thrombopoietin (TPO) signaling pathway	THPO	6.21	122/1277	1.11E−08	918 (2010)	[42, 43]
8	qHTS for inhibitors of ATXN expression: validation	ATXN2	5.94	73/1280	2.67E−07	588,378 (2011)	
9	qHTS assay for the inhibitors of human flap endonuclease 1 (FEN1)	FEN1	5.87	1368/391,275	2.04E−07	588,795 (2011)	
10	AP1 signaling pathway	AP1	5.85	55/10,692	4.90E−5	357 (2006)	[44, 45]

Similarly, we performed Target Enrichment Tree (TET) analysis to get useful clues to underlying targets of phenotypic screening assays. CSgator listed target protein families prioritized by enrichment score (Table 4). The top ranked target family was calcium-activated chloride channel family (ES = 5.90), of which key role was reported in lymph node remodeling by induction of lymphangiogenesis [46]. Among the 40 input compounds, only 2 compounds are known to interact with the targets of the family members in our dataset. It demonstrates the current lack of enough compound-target data even after the integration of 15 publicly available datasets. Therefore, the utility of TET or DET analysis may be limited at the moment compared to HEA analysis. In spite of this limitation, it showed significant enrichment (FDR-adjusted *p* value = 0.00431). We were able to identify other target families potentially associated to lymphangiogenesis. The families related to cytochrome P450 were found frequently within the top 10 ranks (five out of the ten families). It may be associated that oxygen released by oxidoreduction in lymph tissue causes expansion of lymphatic vessels [47]. If we use a larger input set and collect more compound-target dataset, TET analysis may become more useful with better statistical power.

**Table 4:** Target enrichment tree results from lymphangiogenesis hits

Rank	Target family	Target	$Q \otimes H$	Enrichment score	FDR adjusted *p* value
1	CA ACT CL (calcium-activated chloride channel)	ANO1	2	5.90	4.31E−03
3	CYP_3A2 (cytochrome P450 3A2)	Cyp3a2 (Tax ID: 10116)	2	5.30	8.34E−03
4	SLC47 (SLC47 family of multidrug and toxin extrusion transporters)	SLC47A1	2	4.48	2.21E−02
5	Structural (structural protein)	COL1A2	38	4.07	6.33E−31
6	Ca ATPase (calcium ATPase)	ATP2A2	2	3.97	3.81E−02
7	CYP_2E1 (cytochrome P450 2E1)	CYP2E1	5	3.74	4.36E−04
8	CYP_2E (cytochrome P450 family 2E)	CYP2E1	5	3.74	4.57E−04
9	GLY (glycine receptor)	GLRA1	3	3.74	1.02E−02
10	CYP_1B1 (cytochrome P450 1B1)	CYP1B1	3	3.70	1.03E−02
11	CYP_1B (cytochrome P450 family 1B)	CYP1B1	3	3.70	1.06E−02

#### CCA (Compound Cluster Analysis)

**Fig. 2:**
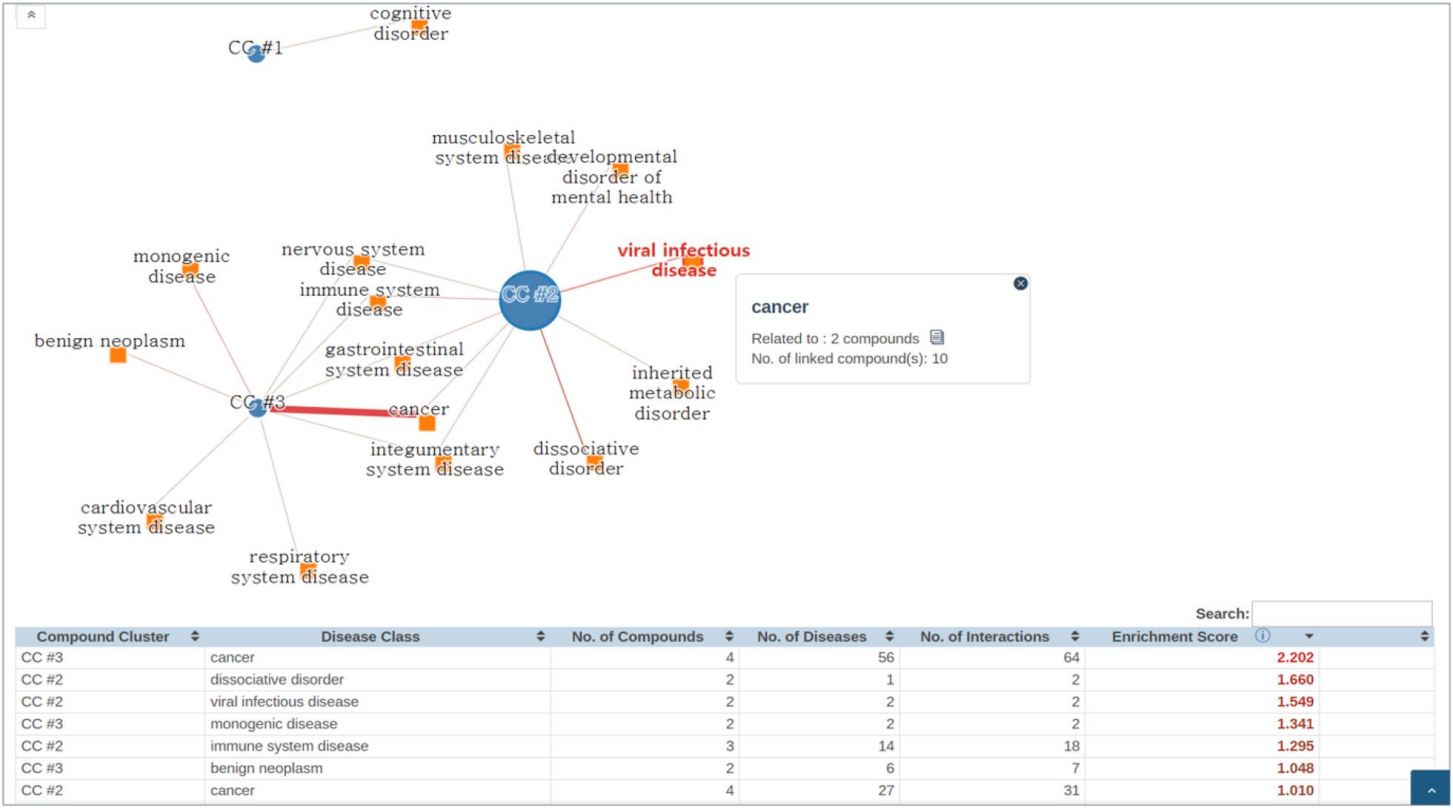
CCA result for the anti-lymphangiogenetic screening hits. CC #1–#3 are the structurally similar clusters of the input compounds generated by k-means clustering (k = 3), which are linked to the relevant DO (Disease Ontology) terms

Certain properties of a compound set may be evident only in structurally similar subgroups. CCA allows identification of enriched features in structurally similar clusters of compounds. In CSgator, we obtained three compound clusters (CC #1–#3) in the 40 input compounds by setting the number of clusters, k = 3. Then, a network of CCs and disease classes is generated (Fig. 2). This network showed the distribution of their original indications, and several notable connections were observed. CC #2 was linked to several diseases including *viral infectious disease*. There are several studies that herpes virus-triggered immune response drives lymphangiogenesis [39, 48, 49]. Both CC #2 and #3 were strongly connected to cancer, which may be expected because inhibition of lymphangiogenesis has emerged as a promising strategy for cancer therapy [47, 50].

## Conclusions

CSgator is a highly comprehensive and integrated analytic system for compound set analysis in terms of targets, bioactivity profiles, structural properties, and disease indications. Such information is crucial to interpret a set of compounds such as high-throughput screening hits, avoid potential side effects or toxicity, and investigate polypharmacology profiles for drug discovery and development. It provides unique functions such as CSEA and CCA, which are not available in other similar tools and databases. It showed that CSgator can give novel clues on drug mode-of-action and the underlying targets for phenotypic screening hits, as shown in the example case of interpreting the anti-lymphangiogenesis screening hits.

## Abbreviations

CSEA: Compound Set Enrichment Analysis
CC: Compound Cluster
CCA: Compound Cluster Analysis
HEA: Hit Enriched Assay
ES: enrichment score
LLR: log likelihood ratio
OR: odds ratio
MDS: multidimensional scaling
HTS: high-throughput screening
SAR: structure–activity relation
InChI: International Chemical Identifier
ID: identification
DB: database
CTD: Comparative Toxicogenomics Database
MeSH: Medical Subject Headings
NIH: National Institutes of Health
NCGC: NCATS Chemical Genomics Center
QED: quantitative estimation of drug-likeness
logP: partition coefficient
Tc: Tanimoto coefficient
GO: gene ontology
DO: Disease Ontology
CAS: Chemical Abstracts Service
qHTS: quantitative high-throughput screening
